# Anakinra in a Preterm Infant with Bronchopulmonary Dysplasia: A Case Report

**DOI:** 10.3390/children13060717

**Published:** 2026-05-22

**Authors:** Simona Fattore, Chiara Tirone, Alessandro Perri, Francesca Paola Fusco, Simonetta Frezza, Milena Tana, Donato Rigante, Davide De Tomaso, Nicoletta Menzella, Alessandra Lio, Francesca Serrao, Stefano Nobile, Andrea Piras, Silvia Baroni, Simonetta Costa, Giovanni Vento

**Affiliations:** 1 Division of Neonatology, Department of Women and Child Health, Fondazione Policlinico Universitario A. Gemelli IRCCS, 00168 Rome, Italy; simona.fattore@guest.policlinicogemelli.it (S.F.);; 2 Department of Women and Child Health Sciences, Child Health Area, Università Cattolica del Sacro Cuore, Largo F. Vito 1, 00168 Rome, Italy; 3 Division of Pediatrics, Department of Women and Child Health, Fondazione Policlinico Universitario A. Gemelli IRCCS, 00168 Rome, Italy; 4 U.O.C. Farmacia, Fondazione Policlinico Universitario A. Gemelli IRCCS, 00168 Rome, Italy; 5 Unit of Chemistry, Biochemistry and Molecular Biology, Fondazione Policlinico Universitario A. Gemelli IRCCS, 00168 Rome, Italy; 6 Department of Basic Biotechnological Sciences, Intensive Care and Perioperative Clinics, Università Cattolica del Sacro Cuore, Largo F. Vito 1, 00168 Rome, Italy; 7 Neonatology and Neonatal Intensive Care Unit, Policlinico Casilino, 00169 Rome, Italy

**Keywords:** bronchopulmonary dysplasia, neonates, anakinra, interleukin-1, personalized medicine, innovative biotechnologies

## Abstract

**Highlights:**

**What are the main findings?**

- Anakinra was administered off-label to a preterm infant with bronchopulmonary dysplasia (BPD), with a temporal association with gradual respiratory improvement.
- Salivary interleukin (IL)-6 and soluble urokinase plasminogen activator receptor levels were explored as non-invasive biomarkers for longitudinal assessment of inflammatory trends during treatment.

**What is the implication of the main findings?**

- IL-1 blockade may represent a potential therapeutic option in selected cases of BPD refractory to standard therapies. Salivary biomarkers may represent a feasible complementary non-invasive approach to describe inflammatory dynamics in neonates.

**Abstract:**

Bronchopulmonary dysplasia (BPD) remains a major complication of extreme prematurity, driven in part by persistent inflammation. Interleukin (IL)-1–mediated signaling plays a central role in sustaining lung injury, making IL-1 blockade a potential therapeutic target. Evidence on the use of anakinra, a recombinant IL-1 receptor antagonist, in neonatal BPD is still limited. We report the case of a female preterm infant (28^+2^ weeks’ gestation, birth weight 800 g, −1.41 zs) affected by BPD requiring prolonged respiratory support. Due to persistent respiratory failure despite standard therapies, off-label treatment with subcutaneous anakinra (5 mg/kg twice daily) was initiated at 150 days of life. Clinical respiratory parameters and exploratory salivary inflammatory biomarkers (IL-6 and soluble urokinase plasminogen activator receptor, suPAR) were longitudinally monitored. Following anakinra initiation, the patient showed a gradual improvement in respiratory parameters, with reduction in oxygen requirement, mean airway pressure, and improved gas exchange. Respiratory support was gradually de-escalated from nasal intermittent positive pressure ventilation to continuous positive airway pressure and subsequently to high-flow nasal cannula. Salivary suPAR levels demonstrated a decreasing trend, while IL-6 showed transient fluctuations, partly associated with intercurrent infections. Treatment was generally well tolerated during the observation period. The infant was discharged on minimal respiratory support, with continued improvement during follow-up. This case suggests a possible role of IL-1 blockade in the modulation of persistent inflammation in BPD with a refractory clinical course, although the observed clinical course may also reflect the natural evolution of the disease. Longitudinal salivary biomarkers may represent a feasible, exploratory, non-invasive approach to describe inflammatory dynamics over time. Larger prospective studies are needed to evaluate the efficacy, safety, and optimal treatment protocols of anakinra.

## 1. Introduction

Despite recent advances in neonatology, which have allowed newborns to survive at very low gestational ages, prematurity remains associated with a high burden of complications, among which bronchopulmonary dysplasia (BPD) represents a major cause of morbidity, refs. [1,2,3]. BPD is a complex disorder with multifactorial etiology, and its incidence is inversely related to gestational age, primarily affecting preterm infants, especially those born before 30 weeks of gestation. The preterm lung, due to its structural and functional immaturity, exhibits several distinctive features, including a reduced capacity to counteract injury induced by oxygen exposure and mechanical ventilation, and increased susceptibility to inflammation [4,5,6]. Inflammation plays a leading role in the development of BPD from intrauterine life onward, with recent evidence indicating that maternal chorioamnionitis may be a significant risk factor [7]. In addition, intrauterine growth restriction (IUGR) has been associated with an increased risk of BPD, likely due to impaired lung growth and altered vascular development in utero [8,9]. After birth, mechanical ventilation, oxygen supplementation, infections, and other intercurrent comorbidities contribute to the establishment and persistence of a pro-inflammatory phenotype [8,9,10,11,12].

Several inflammatory mediators contribute to the lung impairment characteristic of BPD. Acute inflammation, predominant in the early stages, can cause epithelial injury and disrupt the alveolar–capillary structures, while chronic inflammation exacerbates tissue damage, promoting fibrosis as well as reduced regenerative capacity of the lung [7,13]. Interleukin (IL)-1β is a pivotal cytokine active in both acute and chronic inflammation, being involved in the production of other cytokines and inflammatory mediators. Specifically, in BPD, elevated IL-1β levels have been associated with sustained inflammation, which can further impair lung tissue and interfere with normal organ maturation [14,15,16]. In addition to IL-1β, IL-1α may also contribute to lung inflammation, as it can be released by damaged epithelial cells and act locally as an “alarmin”, initiating and amplifying the inflammatory response at the tissue level. Both cytokines signal through the same IL-1 receptor [17].

Given the central role of IL-1 in sustaining inflammation, targeting this cytokine with a recombinant non-glycosylated form of the human IL-1 receptor antagonist, named anakinra, has been proposed as a potential therapeutic strategy in moderate to severe forms of BPD [18,19]. Anakinra has been traditionally used in the management of many inherited autoinflammatory diseases occurring in children and adults, characterized by periodic fevers with elevated acute-phase proteins during flares and linked to excessive bioactivity of IL-1 [20]. However, anakinra has recently been studied in the neonatal setting, given its ability to modulate inflammation, making it a potential therapeutic alternative for inflammation-related conditions such as BPD [21,22]. However, clinical evidence supporting the use of IL-1 blockade in neonatal BPD remains limited, and current knowledge is largely based on preclinical data and small clinical observations.

In parallel, there is increasing interest in identifying biomarkers that may help monitor disease activity and inflammatory burden over time, particularly through non-invasive approaches that are suitable for fragile populations such as preterm infants. In adult populations, circulating biomarkers such as interleukin-6 (IL-6) and soluble urokinase plasminogen activator receptor (suPAR) have been used to reflect systemic inflammation and disease severity. However, their role in neonatal populations, and specifically in bronchopulmonary dysplasia, remains largely unexplored [23,24].

In this case report we present the history of a preterm infant with BPD with a refractory clinical course who was treated with off-label anakinra, with a focus on the temporal clinical evolution and on the feasibility of longitudinal monitoring using salivary inflammatory biomarkers.

The aim of this report is to describe the clinical course associated with IL-1 blockade and to explore the feasibility of longitudinal monitoring using exploratory salivary inflammatory biomarkers.

## 2. Case Report

A female neonate was born in an outside hospital at 28^+2^ weeks of gestation with a birth weight of 800 g. At birth, she was small for gestational age with a z-score of −1.41, based on Intergrowth-21 charts [25].

The neonate was delivered by urgent caesarean section due to IUGR and abnormal Doppler flow. Antenatal corticosteroid prophylaxis with a complete course of betamethasone had been administered. At birth, she had cardiorespiratory depression, requiring orotracheal intubation. Apgar scores were 4 and 6 at 1 and 5 min, respectively.

The clinical course was characterized by severe respiratory distress syndrome, which required four doses of surfactant, pulmonary hypertension needing inhaled nitric oxide administration, and hemodynamically significant patent ductus arteriosus.

The infant experienced one episode of late-onset neonatal sepsis (LONS) and two episodes of pneumonia at the referring center. The causative organisms included Staphylococcus aureus for LONS and Streptococcus hemolyticus and Enterobacter cloacae for pneumonia. The diagnosis of LONS was based on a positive blood culture associated with compatible clinical signs; the diagnosis of pneumonia was based on positive bronchoalveolar lavage (BAL) cultures, in association with worsening respiratory status and compatible radiological or lung ultrasound findings. Overall, she required 30 days of mechanical ventilation and subsequently non-invasive respiratory support with supplemental oxygen until the time of transfer.

A diagnosis of BPD (Jensen grade II) [26] had been established at the referral center at PMA 36 weeks: at that time, the infant was receiving CPAP at 10 cmH_2_O with FiO_2_ approximately 0.50. The infant received diuretics, caffeine, and three courses of dexamethasone. The transfer to our hospital occurred at 48^+2^ weeks postmenstrual age (PMA) to evaluate a possible tracheostomy.

Upon admission to our neonatal intensive care unit, the patient received nasal intermittent positive pressure ventilation (n-IPPV) with the following parameters: PIP/PEEP 32/15 cm H_2_O, FiO_2_ 0.50, respiratory rate 25 bpm, Ti: 0.6 s, with the aim of achieving SpO_2_ and pCO_2_ values within the target range (92–95% and 50–60 mmHg, respectively). After 10 days of inability to reduce FiO_2_ and intensity of respiratory support, given the severity of her respiratory condition, off-label treatment with anakinra was started on day 150 of life (PMA 49^+5^ weeks) at a dose of 5 mg/kg twice daily, administered subcutaneously. The decision to initiate anakinra was made in the context of persistent severe respiratory failure despite optimized supportive care and three prior courses of systemic corticosteroids. At the time of transfer, the patient had been referred for evaluation of possible tracheostomy, reflecting the severity and refractoriness of her condition; however, this option was not favored by the parents. In the absence of further conventional therapeutic options and considering the underlying inflammatory component of BPD, IL-1 blockade was therefore considered a rescue anti-inflammatory strategy.

The dosing regimen was selected based on available pediatric experience and limited neonatal reports in hyperinflammatory conditions, where similar weight-based dosing has been used. The twice-daily regimen was preferred over once-daily administration with the aim of maintaining stable drug levels throughout the day and as a safety measure, considering the lack of established neonatal dosing protocols in this setting. Given the renal clearance of anakinra and the potential variability in neonatal pharmacokinetics, close clinical and laboratory monitoring was performed throughout treatment [21].

**Figure 1:**
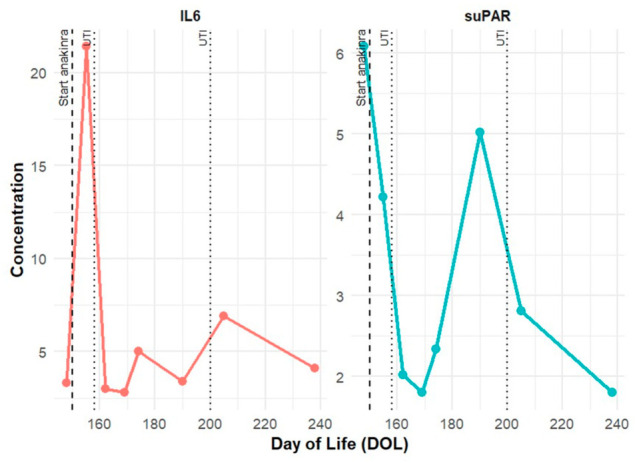
Longitudinal trends of salivary interleukin-6 (IL-6, pg/mL) and soluble urokinase plasminogen activator receptor (suPAR, ng/mL) concentrations measured at weekly intervals before and during treatment. Day of life (DOL) is reported on the *X*-axis. The dashed vertical line indicates the initiation of anakinra therapy (DOL 150), while dotted vertical lines indicate episodes of urinary tract infection (UTI) occurring during follow-up (DOL 158 and DOL 200). UTI diagnosis refers to the day of clinical and microbiological confirmation. Values below the limit of detection for suPAR were imputed as the detection threshold (1.8 ng/mL). No episodes of proven sepsis occurred during the period of sample collection.

Salivary samples were collected weekly, starting before the initiation of anakinra and throughout the treatment period. IL-6 and soluble urokinase plasminogen activator receptor (suPAR) concentrations were measured to evaluate longitudinal changes using immunoassays validated for clinical use and performed on automated Siemens Atellica analyzers (Siemens Healthcare Diagnostics Inc., Tarrytown, NY, USA). Although these immunoassays are validated for clinical use, they have not been specifically validated for neonatal salivary specimens. Specifically, IL-6 was measured using the chemiluminescent immunoassay (CLIA) Atellica IM Interleukin-6 kit, while suPAR was determined using the immunoturbidimetric suPARnostic^®^ TurbiLatex (ViroGates, Birkerød, Denmark) assay on the Siemens Atellica CH platform. At baseline (day of life 148), suPAR and IL-6 levels were 6.08 ng/mL and 3.3 pg/mL, respectively. After initiation of anakinra on day 150 of life, suPAR levels showed a general decreasing trend over time, with intermittent fluctuations, reaching values below 1.8 ng/mL at day 169 and again at day 238. IL-6 levels showed a more variable pattern, with transient peaks (e.g., 21.4 pg/mL at day 155, preceding the diagnosis of urinary tract infection made at day 158), followed by subsequent decreases (Figure 1). The infant was fed predominantly with formula milk. Saliva samples were preferentially collected before feeding; however, complete standardization of sampling timing was not always feasible. Although exposure to breast milk was limited, a potential influence on cytokine levels cannot be entirely excluded. Total saliva was collected using a soft plastic aspirator as it flowed onto the front of the floor of the mouth. After collection, each saliva sample was immediately diluted 1:1 (*v*/*v*) with a 0.2% aqueous solution of 2,2,2-trifluoroacetic acid (TFA) and subsequently stored at −80 °C.

**Figure 2:**
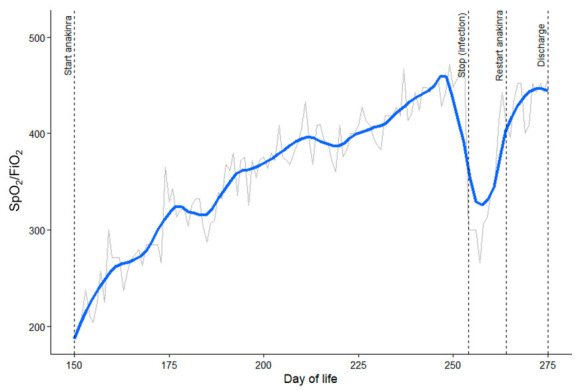
The daily SpO_2_/FiO_2_ (S/F) ratio was calculated using mean daily SpO_2_ and mean daily FiO_2_ values in the patient treated with anakinra. The *X*-axis represents the day of life (DOL), while the *Y*-axis the daily SpO_2_/FiO_2_ ratio. Vertical dashed lines indicate initiation of anakinra therapy, temporary discontinuation of anakinra due to intercurrent viral respiratory infection, reintroduction of therapy with anakinra, and hospital discharge.

**Figure 3:**
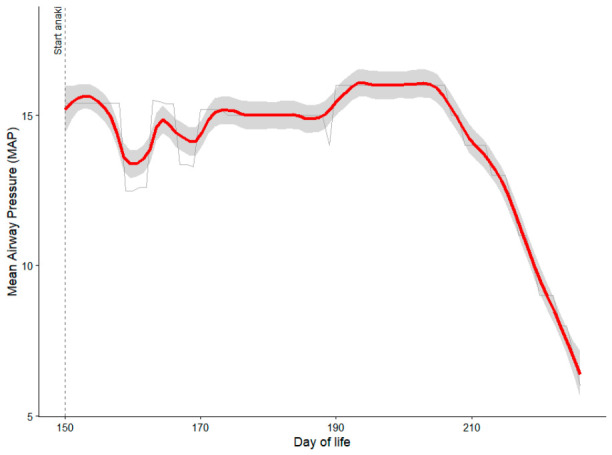
Daily mean airway pressure (MAP) in the patient treated with anakinra is shown over time from day 150 to day 226 of life, corresponding to the period of nasal-intermittent positive pressure ventilation (n-IPPV) and continuous positive airway pressure (CPAP) support.

During the treatment period, daily clinical monitoring included mean airway pressure (MAP) and oxygen requirement (FiO_2_), as well as blood gases analysis. Over the course of treatment, the patient demonstrated gradual improvement in respiratory stability. FiO_2_ requirements progressively decreased, resulting in an improved SpO_2_/FiO_2_ ratio (Figure 2), and MAP was progressively reduced (Figure 3). Given the use of different modes of respiratory support, the SpO_2_/FiO_2_ ratio was interpreted as a descriptive longitudinal parameter.

**Figure 4:**
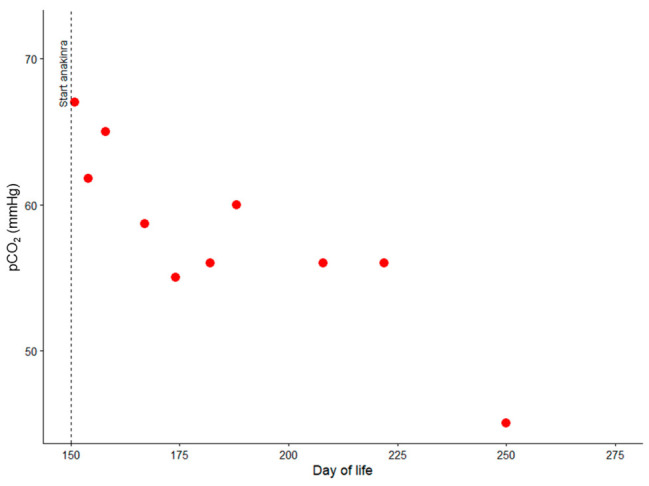
Changes in capillary pCO_2_ levels over time during anakinra treatment.

Furthermore, gas exchange parameters gradually improved (Figure 4). Respiratory support was progressively de-escalated: the infant was supported with n-IPPV from day 150 to day 173 of life, followed by continuous positive airway pressure (CPAP) from day 174 to day 226, and subsequently transitioned to high-flow nasal cannula (HFNC).

Complete blood counts with differential, renal function, and liver function tests were monitored serially during treatment, approximately every 10 days during the first six weeks and less frequently thereafter. A mild transient neutropenia (absolute neutrophil count 1270/mm^3^) was observed approximately one month after initiation of anakinra. As the patient remained clinically stable and neutropenia was mild, treatment was neither interrupted nor dose-reduced, and only the subsequent laboratory monitoring was anticipated. Neutropenia resolved spontaneously without clinical consequences.

No other clinically significant hematological abnormalities were detected. Creatinine levels and transaminases remained within normal ranges throughout the treatment period. No injection-site reactions were observed. Infection surveillance was based on clinical assessment and routine microbiological investigations when indicated.

In addition, the infant experienced two episodes of urinary tract infection after the initiation of anakinra, which were not associated with significant clinical deterioration and during which anakinra therapy was continued without modification. Since a postmenstrual age of 61^+5^ weeks, the infant breathed spontaneously while awake, alternating with HFNC support at 7 L/min and FiO_2_ 0.21 during sleep. After approximately three weeks, respiratory support had to be resumed during wakefulness due to an intercurrent viral respiratory infection. During this infection, anakinra was discontinued and later resumed at a reduced once-daily dose (5 mg/kg/day), as a precautionary measure to balance potential immunosuppressive effects while maintaining anti-inflammatory activity. The infant was discharged at 276 days of life, PMA 67^+5^ weeks, on HFNC 8 L/min, FiO_2_ 0.21. Follow-up evaluations showed progressive improvement in respiratory parameters, allowing discontinuation of respiratory support during awake periods. Anakinra therapy was discontinued approximately one month after discharge, after a period of maintained clinical stability, with the aim of supporting this stability during a transitional phase of disease evolution under close outpatient monitoring.

**Figure 5:**
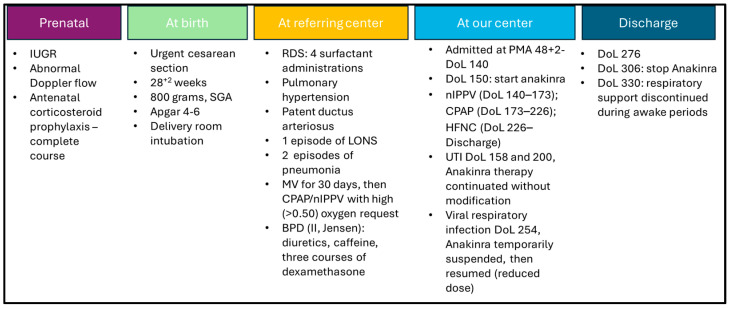
Timeline of major clinical events. IUGR: Intrauterine growth restriction. SGA: Small for gestational age. RDS: Respiratory distress syndrome. LONS: Late onset neonatal sepsis. MV: Mechanical ventilation. CPAP: Continuous Positive Airway Pressure. nIPPV: nasal intermittent positive pressure ventilation. BPD: Bronchopulmonary Dysplasia. PMA: Postmenstrual Age. DoL: Day of Life. HFNC: High-Flow Nasal Cannula.

**Figure 6:**
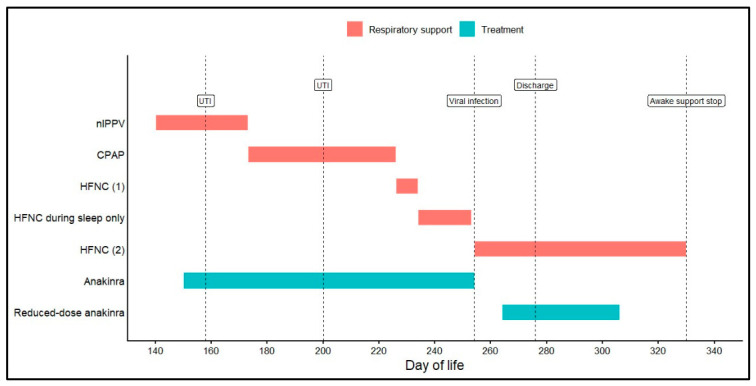
Graphical timeline summarizing the clinical course after admission to our center. Horizontal bars represent respiratory support modalities and anakinra treatment over time. Vertical dashed lines indicate major clinical events. nIPPV: nasal intermittent positive pressure ventilation. CPAP: Continuous Positive Airway Pressure. HFNC: High-Flow Nasal Cannula. UTI: Urinary tract infection.

A detailed timeline of clinical events is provided in Figure 5 and Figure 6.

## 3. Discussion

IL-1 antagonists have shown efficacy in autoinflammatory conditions, supporting their potential relevance in diseases characterized by dysregulated inflammation such as BPD.

The remarkable progress with IL-1 antagonists in treating hereditary inflammasome-based disorders has offered new hope for several patients with non-hereditary autoinflammatory conditions having a multifactorial background: indeed, the effectiveness of IL-1 blockade is transforming the management of complex diseases characterized by aberrant IL-1 signaling without autoreactive T-cells or autoantibody production. To date, long-term blockade of IL-1 has become a valuable weapon to manage cryopyrin-associated periodic syndrome, Still’s disease, and refractory Kawasaki disease [27,28,29].

An increased production of IL-1β has been also demonstrated by multiple studies in both mother and fetus during chorioamnionitis, including the amniotic fluid, placenta and chorioamniotic membranes [30]. Furthermore, BPD is underpinned by a dramatic surge in pulmonary inflammation, and efficacious treatments remain limited. Systemic corticosteroids are widely used in selected cases, with careful consideration of the balance between potential respiratory benefits and known risks, including possible effects on alveolar growth. A significant adverse effect on growth has been demonstrated by the low postnatal head circumference of infants treated with high-dose dexamethasone [31]. The best-known mediator of pulmonary inflammation is IL-1β, playing also a crucial role in the inflammatory damage to the brain, eye and gut of premature infants [32]. Recent clinical evidence further supports the involvement of the IL-1 pathway in BPD, as altered circulating levels of IL-1 family cytokines have been associated with BPD in extremely preterm infants [33].

Our report refers to a preterm infant with BPD treated with anakinra, a recombinant IL-1 receptor antagonist that displays significant immunomodulatory activity: the patient showed a gradual respiratory improvement, achieving spontaneous breathing during awake periods. This clinical observation may suggest the potential role of targeting IL-1 in modulating the persistent inflammation characteristic of BPD with a refractory clinical course in preterm infants.

IL-1β is a central cytokine in both acute and chronic inflammation and has been implicated, in preclinical models, in tissue injury and impaired alveolar development [14,32]. Anakinra exerts its effects by blocking the IL-1 receptor type 1, thereby antagonizing the activity of both IL-1α and IL-1β. While IL-1β has been more extensively implicated in the pathogenesis of BPD, IL-1α, acting as an alarmin released from damaged cells, may also contribute to local pulmonary inflammation. Therefore, the potential clinical effects are likely related to the combined inhibition of IL-1-mediated signaling rather than to a single cytokine [16,34,35].

By blocking IL-1 signaling, anakinra may attenuate the inflammatory cascade, potentially reducing ongoing lung injury and contributing to the stabilization of respiratory function. Preclinical models related to BPD have shown that the use of anakinra in this population marked by an oversensitive innate immune system can curb the inflammatory cascade and reduce lung injury [34,36,37]. Although inflammatory injury in BPD is classically most prominent in the early postnatal period, increasing evidence suggests that the IL-1 signaling axis may remain dysregulated in a subset of infants with severe disease. In these cases, persistent inflammatory activity may coexist with structural remodeling rather than representing strictly sequential disease phases. Longitudinal studies have demonstrated sustained alterations in IL-1 family cytokines and imbalance between IL-1β and IL-1 receptor antagonist extending beyond the early neonatal period, with associations with disease severity even at later postmenstrual ages [34].

Recent studies and small case series have suggested that anakinra is safe and feasible in neonates with hyperinflammatory conditions, although data specifically related to BPD are extremely limited [21,22]. A recent case series has reported the use of anakinra in heterogeneous hyperinflammatory conditions, including a preterm infant with bronchopulmonary dysplasia. In this report, IL-1 blockade was considered a potential option in selected refractory conditions, particularly when first-line anti-inflammatory therapies failed to adequately control disease manifestations [21].

The improvement observed in our patient was temporally associated with the initiation of anakinra and is consistent with the biological plausibility of IL-1-targeted therapy in BPD. However, several confounding factors should be considered, including prior exposure to systemic corticosteroids, resolution of intercurrent infections, and optimization of respiratory support. In addition, with advancing postmenstrual age, infants with BPD and a refractory clinical course may show gradual clinical improvement as part of the natural course of the disease. Therefore, the present observation should be interpreted as hypothesis-generating, and no causal relationship can be established.

To the best of our knowledge, this case adds to the very limited available evidence on the use of anakinra in preterm infants with BPD. In addition, it provides an exploratory description of longitudinal salivary inflammatory markers in this context, an area that remains largely under-investigated. Anakinra was initiated at 150 days of life (PMA 49^+5^ weeks) and continued for approximately 4 months. This therapy was temporarily discontinued during an intercurrent respiratory infection and later resumed at a reduced dose, reflecting the need for careful monitoring of infections along with anakinra administration.

The progressive reduction of respiratory support and subsequent possibility of discontinuing anakinra may be consistent with a potential contribution of the treatment. However, this observed response may also have been influenced by the natural lung maturation and supportive care, highlighting that observations based on a single case do not allow a causal inference. Safety considerations are particularly relevant in this context, given the immunomodulatory effects of IL-1 blockade. In our patient, no major adverse events were observed during treatment, although intercurrent infections occurred and the contribution of anakinra to infection risk cannot be excluded. More broadly, the potential effects of IL-1 modulation on developing organ systems remain incompletely understood in preterm infants and warrant further investigation. A mild transient neutropenia was observed during treatment, occurring approximately one month after initiation and resolving spontaneously without clinical consequences. Although this finding is consistent with known potential side effects of anakinra, no persistent or clinically relevant hematological abnormalities were detected in this case. In addition to hematological monitoring, renal and hepatic function remained stable, and no injection-site reactions were observed.

In addition to clinical observations, we conducted an exploratory longitudinal assessment of salivary IL-6 and suPAR. Direct quantification of IL-1 was not performed due to its short half-life, low stability, and marked temporal variability, which are particularly challenging in neonatal samples and in salivary matrices, limiting its reliability as a longitudinal biomarker in this setting [24,38]. The temporal trends observed in our patient suggested a dynamic modulation of the inflammatory environment over time, with variations that may parallel changes in disease activity and response to treatment, although infections and other intercurrent events must be taken into account.

Although nonspecific to IL-1 signalling, IL-6 and suPAR are functionally associated with inflammatory pathways that may be influenced, among other stimuli, by IL-1-mediated signaling. IL-6 acts as a downstream mediator of IL-1 activity: IL-1 acts as a potent inducer of IL-6, positioning itself upstream in an amplifying inflammatory cascade: binding of IL-1 to its receptor activates multiple intracellular signaling pathways that converge on IL-6 gene transcription. This hierarchical relationship has important therapeutic implications, as IL-1 blockade reduces IL-6 levels and its downstream mediators [39,40].

SuPAR is the circulating soluble form of the urokinase-type plasminogen activator receptor (uPAR). Although its production is not directly induced by IL-1, suPAR is a biomarker of inflammation reflecting the overall level of immune activation. Circulating levels correlate with those of IL-1, but, unlike IL-1, suPAR is more stable and less influenced by circadian variation [41]. SuPAR is considered a prognostic biomarker reflecting the magnitude of systemic inflammation and immune activation and may provide additional information on disease severity and response to therapy [35,41]. In this context, their longitudinal behavior may reflect dynamic inflammatory changes over time. While these findings are limited by the single-case design and should be interpreted with caution, they support the feasibility of using salivary biomarkers as complementary and minimally invasive tools to describe longitudinal inflammatory trends in preterm infants with BPD. It should be acknowledged that both IL-6 and suPAR are not validated biomarkers in neonatal bronchopulmonary dysplasia, and no established reference ranges exist in this population. Moreover, although clinically validated immunoassays were used, these assays have not been specifically validated for neonatal salivary specimens. In addition, IL-6 is highly susceptible to fluctuations related to intercurrent infections, which likely contributed to the observed variability. Therefore, the longitudinal salivary findings should be interpreted as an exploratory, descriptive observation of inflammatory trends rather than as indicators of treatment response or efficacy.

This experience supports the rationale for further studies on IL-1 blockade in moderate to severe BPD, at approximately 36 weeks PMA. Although anakinra was initiated at a later stage in our patient, the observed temporal association with clinical improvement suggests that inflammatory pathways may remain active beyond the early postnatal period. This raises the possibility that earlier intervention, at a stage when inflammatory mechanisms may be more prominent and structural lung damage less established, could potentially result in a greater therapeutic impact. However, this hypothesis remains speculative and hypothesis-generating, as anakinra was initiated relatively late in the present case (PMA 49^+5^ weeks), and therefore no direct conclusions regarding the optimal timing of IL-1 blockade can be drawn from this single-case report. Prospective, possibly multicenter, studies are warranted to better evaluate the optimal timing, dosing, and duration of therapy, as well as the overall safety and long-term outcomes of anakinra in this specific context. The identification of biomarkers predicting response to anakinra may also help identify neonates who could most benefit from targeted anti-cytokine therapy.

## 4. Conclusions

The use of anakinra in this very preterm neonate with BPD was feasible and generally well tolerated and it was temporally associated with gradual respiratory improvement. While evidence from a single case is inherently limited, our report adds to the growing interest in targeted anti-inflammatory interventions for BPD and supports further investigation of IL-1 blockade as a potential therapeutic strategy.

## Data Availability

The original contributions presented in this study are included in the article. Further inquiries can be directed to the corresponding authors.

## Abbreviations

The following abbreviations are used in this manuscript:

BPD	Bronchopulmonary dysplasia
IL	Interleukin
suPAR	Soluble urokinase plasminogen activator receptor
IUGR	intrauterine growth restriction
SGA	Small for gestational age
LONS	Late-onset neonatal sepsis
BAL	Bronchoalveolar lavage
PMA	Postmenstrual age
n-IPPV	Nasal intermittent positive pressure ventilation
PIP	Peak inspiratory pressure
PEEP	Positive end-expiratory pressure
FiO_2_	Fraction of inspired oxygen
SpO_2_	Oxygen saturation
pCO_2_	Partial pressure of carbon dioxide
MAP	Mean airway pressure
CPAP	Continuous positive airway pressure
HFNC	High-flow nasal cannula
DOL	Day of life
UTI	Urinary tract infection
